# Exploring the Connection Between Ultra-Processed Foods and Breast Cancer: From Adiposity to Inflammation and Beyond

**DOI:** 10.3390/ijms27073173

**Published:** 2026-03-31

**Authors:** Rodrigo Soares Pereira Lima, Jheniffer da Silva Sousa, Dallyla Jennifer Morais de Sousa, Jorddam Almondes Martins, Irislene Costa Pereira, Rebeca Lima Monteiro, Maria Shelda de Oliveira Neres, Juliana Soares Severo, Felipe Cavalcanti Carneiro da Silva, Moisés Tolentino Bento da Silva, Francisco Leonardo Torres-Leal

**Affiliations:** 1 Metabolic Diseases, Exercise and Nutrition Research Group (DOMEN), Laboratory of Metabolic Diseases Glauto Tuquarre, Department of Biophysics and Physiology, Center for Health Sciences, Federal University of Piauí, Teresina 64049-550, PI, Brazil; rodrigo.soares@ufpi.edu.br (R.S.P.L.); jhenyss@hotmail.com (J.d.S.S.); dallylajenniferms@gmail.com (D.J.M.d.S.); jorddamalmondes@ufpi.edu.br (J.A.M.); irisllenycx@hotmail.com (I.C.P.); nutrirebecamonteiro@gmail.com (R.L.M.); mshelda123@ufpi.edu.br (M.S.d.O.N.); 2 Laboratory of Toxicological Genetics (LAPGENIC), Federal University of Piauí, Teresina 64049-550, PI, Brazil; felipebio@ufpi.edu.br; 3 Laboratory of Physiology, Center for Drug Discovery and Innovative Medicines (MedInUP)—RISE-Health: Health Research Network, Department of Immuno-Physiology and Pharmacology, School of Medicine and Biomedical Science—University of Porto (ICBAS—UP), 2284050-313 Porto, Portugal; mtsilva@icbas.up.pt

**Keywords:** ultra-processed foods, obesity, breast cancer, nutrition, eating patterns

## Abstract

In this narrative review, we synthesize current epidemiological and mechanistic evidence on the association between ultra-processed food (UPF) consumption, obesity, and breast cancer (BC) risk. The global increase in UPF intake has been linked to substantial metabolic alterations, including weight gain, insulin resistance, low-grade chronic inflammation, and hormonal imbalances—factors that contribute to a tumor-promoting microenvironment. Given the established role of obesity in breast carcinogenesis, understanding the intermediary role of UPFs is critical. Articles were selected from PubMed, SciELO, and ScienceDirect databases using descriptors related to ultra-processed foods, obesity, and breast cancer. The synthesis of current evidence supports the view that excessive UPFs consumption represents a modifiable and preventable risk factor for obesity and BC, underscoring the need for integrated strategies in dietary guidance, public health policy, and cancer prevention.

## 1. Introduction

**Figure 1:**
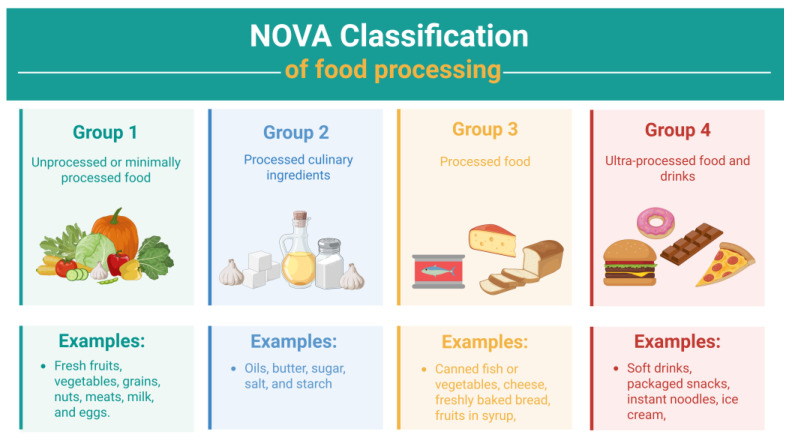
NOVA classification system. Created in BioRender. Torres-Leal, L. (2026) https://BioRender.com/kixyxa2.

The progressive industrial transformation of food production has markedly reshaped dietary behaviors worldwide. Ultra-processed foods (UPFs) now represent a considerable proportion of total energy intake across both high- and middle-income nations. Parallel to this dietary shift, the prevalence of obesity, metabolic disturbances, and diet-related malignancies has increased globally, raising concerns about the potential contribution of UPFs to disease development. To better characterize food processing levels, the NOVA classification system was introduced in 2009 by Brazilian researchers, categorizing foods according to the degree and purpose of industrial processing [1]. This framework distinguishes four groups: unprocessed or minimally processed foods, processed culinary ingredients, processed foods, and ultra-processed foods [2]. Figure 1 summarizes the NOVA classification system [2].

Ultra-processed foods (UPFs) consist of industrial formulations predominantly manufactured from substances not commonly used in domestic kitchens, often derived from multiple sequential processing steps. These processes may include the extraction and recombination of food components, along with the addition of cosmetic additives and packaging strategies aimed at extending shelf life [2]. Formulated to enhance palatability, convenience, and durability, UPFs have progressively expanded their presence in the global food supply. In several high-income countries, including the United States, Canada, and the United Kingdom, UPFs contribute to more than half of total daily energy intake [3,4,5]. In the UK specifically, greater reliance on UPFs has been associated with reduced engagement in home cooking practices and structural constraints such as limited time availability [6]. Although lower than in these countries, the contribution of UPFs to total caloric intake in Brazil reached 19.7% between 2017 and 2018, with national Household Budget Survey data demonstrating a consistent upward trend in household purchases [7].

This narrative review examines consumption trends in Brazil and worldwide and explores the relationship between UPF intake and chronic non-communicable diseases, particularly obesity and breast cancer (BC) [8,9,10,11,12]. Accumulating epidemiological evidence, including large prospective cohorts and dose–response meta-analyses, has reported positive associations between UPF consumption and adverse health outcomes such as obesity, BC, and all-cause mortality [13,14,15,16]. For example, each 10% increment in daily energy intake derived from UPFs has been associated with increased risks of obesity (7%), overweight (6%), combined overweight/obesity (3%), and abdominal obesity (5%) [14].

The relationship between UPFs and excess adiposity is especially relevant given the established role of obesity in BC pathophysiology [17,18]. Dietary patterns characterized by high energy density and low intake of fiber, antioxidants, and micronutrients contribute to progressive weight gain, particularly among physically inactive individuals [19]. The resulting expansion of adipose tissue promotes a chronic low-grade inflammatory state that may create a pro-tumorigenic microenvironment, thereby facilitating BC development and progression [20].

Excess adiposity functions as a metabolic platform for tumor development through mechanisms involving hyperglycemia, hyperinsulinemia, and adipocyte hypertrophy. The latter, unaccompanied by adequate angiogenesis, leads to tissue hypoxia, fibrosis, and inflammation. Additionally, adipose tissue may serve as an energy reservoir for tumors and contributes to estrogen synthesis via hormonal signaling pathways [21].

While evidence linking UPFs to obesity and BC continues to grow, the literature remains fragmented. Despite the growing number of observational studies, the biological and mechanistic pathways linking UPFs consumption to breast cancer—whether directly or mediated through adiposity—remain insufficiently integrated. To date, no narrative review has comprehensively synthesized the epidemiological evidence, metabolic underpinnings, and regulatory dimensions of this relationship.

In light of the escalating global prevalence of obesity and its recognized contribution to breast cancer (BC) development, together with growing evidence implicating UPFs in adverse metabolic and oncologic outcomes, this review seeks to integrate and critically examine the available literature on the interplay between UPF consumption, excess adiposity, and breast carcinogenesis. By consolidating current evidence, we aim to provide a conceptual framework that may support more effective prevention and therapeutic approaches targeting these interconnected conditions.

## 2. Methods and Materials

The present narrative review was designed to synthesize and critically interpret the existing literature regarding the association between ultra-processed food (UPFs) consumption, metabolic disturbances, inflammatory pathways, and breast cancer development. Because the available studies differ substantially in terms of methodological design, outcomes assessed, and mechanistic focus, conducting a formal systematic review or meta-analysis was not considered appropriate for the scope of this work. Instead, a narrative approach was adopted to allow the integration of evidence derived from experimental, translational, and clinical research.

The evidence was examined qualitatively, prioritizing prospective human studies whenever available. Observational studies, meta-analyses, and mechanistic investigations using experimental models were also considered to provide complementary insights into the biological mechanisms potentially linking dietary exposures to breast cancer. When discrepancies among studies were identified, findings were interpreted by considering methodological quality, biological plausibility, and contextual relevance within the broader scientific literature. As this work aims to provide a conceptual and integrative overview, a formal risk-of-bias assessment tool was not applied; this limitation is acknowledged as an inherent characteristic of the narrative review methodology.

A comprehensive literature search was performed in the databases PubMed/MEDLINE, Scopus, and ScienceDirect from their inception until February 2026. No restrictions regarding language or publication year were imposed in order to capture the widest possible range of relevant publications. Furthermore, the reference lists of selected articles were manually screened to identify additional studies that might have been missed during the initial search.

The search strategy combined Medical Subject Headings (MeSH) and free-text terms related to ultra-processed foods, the NOVA classification system, obesity, breast cancer, inflammation, gut microbiota, epigenetic mechanisms, and dietary patterns. These terms were combined using Boolean operators to identify studies investigating potential epidemiological and biological relationships between dietary exposures and breast cancer risk or progression.

Studies were eligible for inclusion if they consisted of original research conducted in humans, animal models, or cell-based experimental systems that reported mechanistic, physiological, metabolic, or clinical evidence related to ultra-processed food consumption or breast cancer biology. Review articles and meta-analyses were also considered when they provided relevant theoretical background or contextual support for the topics discussed. Conference abstracts, expert opinion papers, and studies not directly addressing the research question were excluded.

The selection procedure involved an initial screening of titles and abstracts to identify potentially relevant publications, followed by a full-text evaluation of eligible articles. The final body of literature included in this narrative synthesis was determined based on the relevance of each study to the relationship between ultra-processed food intake, metabolic dysregulation, and breast cancer.

For analytical purposes, the included studies were organized into thematic categories, including preclinical investigations, observational or clinical studies involving human populations, and review articles providing complementary evidence. Given the narrative nature of this work, no standardized framework for methodological quality assessment or risk-of-bias evaluation was applied.

## 3. Ultra-Processed Foods: Contextualization of Consumption in Brazil and in the World

Ultra-processed foods (UPFs) currently constitute a substantial portion of contemporary dietary patterns and encompass a wide spectrum of industrially manufactured products. These include sugar-sweetened beverages, packaged sweet or savory snacks, confectionery items, baked goods such as cookies and cakes, spreads and margarines, sweetened breakfast cereals, ready-to-eat meals, processed meat products (e.g., nuggets, sausages, burgers), instant soups, and packaged pasta dishes and desserts. Despite the operational definition proposed by the NOVA framework, ongoing discussion persists regarding the classification of certain products—such as packaged whole-grain breads, fruit-flavored yogurts, fortified juices, and plant-based meat substitutes—within the UPF category [22].

Since the late twentieth century, particularly from the 1980s onward, global sales and consumption of UPFs have risen markedly, reflecting a broad dietary transition toward industrially processed food products [23]. However, substantial heterogeneity exists across regions, likely influenced by economic development, regulatory environments, cultural norms, market expansion strategies, and shifts in food purchasing practices. In middle-income countries, UPFs currently contribute between approximately 5% of total daily energy intake in China and nearly 30% in Mexico [24,25]. Even so, rapid growth in UPF sales has been documented across parts of Asia, the Middle East, and Africa, signaling an accelerating nutrition transition in these regions [26,27].

Patterns of UPFs consumption are not evenly distributed within populations. Higher intake levels have been associated with particular sociodemographic and behavioral characteristics, including younger age, female sex, lower socioeconomic and educational status, solitary living arrangements, higher body mass index (BMI), lower levels of physical activity, and prolonged screen exposure during meals [22]. From a nutritional standpoint, dietary patterns rich in UPFs are typically energy-dense and characterized by elevated intakes of free sugars, total and saturated fats, alongside comparatively lower consumption of protein, dietary fiber, and essential micronutrients [27,28,29].

The industrial formulation of these products is strategically optimized for profitability, relying on low-cost ingredients, extended shelf life, convenience, and enhanced palatability. Combined with aggressive marketing practices, these characteristics confer a substantial competitive advantage to ultra-processed foods across global food markets [2]. In Brazil, the predominance of UPFs in mass media has been documented through the analysis of 432 h of programming on major free-to-air television channels. Among 7991 advertisements, 14.2% (0.89 ads/channel per hour) were food-related, and 91% (0.60 ads/channel per hour) of these promoted UPFs, particularly soft drinks, alcoholic beverages, and fast-food products [30]. Such widespread media exposure represents a significant public health concern, given its capacity to shape dietary behaviors and influence long-term health outcomes.

Digital platforms have further amplified the reach and sophistication of UPF marketing strategies. Social media applications such as Twitter, Instagram, and Facebook facilitate highly interactive campaigns that integrate videos, images, music, and gamified content. In Brazil, approximately 62 million individuals access Facebook on a daily basis, and 6% of the platform’s most liked pages are associated with UPF brands. Notably, among the ten most-followed pages by Brazilian users, two are related to UPFs. Marketing approaches are characterized by high levels of user engagement, including more than 550,000 comments on McDonald’s Facebook page, extensive use of video-based content (93%), promotional incentives (68.8%), and celebrity endorsements (62.5%) [31].

Dietary data reveal consistent patterns of UPFs consumption in Brazil. The most consumed subgroups include margarine, loaf bread, hot dogs and hamburgers, soft drinks, sausages, processed meats, packaged desserts, and sweet snacks or cookies [32]. Consumption of five or more UPFs subgroups declines linearly with age and is inversely associated with female sex and higher educational attainment. Between 2002 and 2003 and 2017 and 2018, UPFs increased from 12.6% to 18.4% of total caloric intake, while the share of unprocessed or minimally processed foods and culinary ingredients fell by 3.8% and 3.5%, respectively. The South and Southeast regions recorded the highest caloric contributions from UPFs [7].

In the 2017–2018 survey, Brazilians aged 10 years and older had an average daily energy intake of 1754.61 kcal. Of this total, 53.25% came from unprocessed or minimally processed foods, 15.78% from processed culinary ingredients, 11.28% from processed foods, and 19.69% from UPFs. Rice, beef, beans, and poultry were the main contributors within the unprocessed food group, whereas fruits, milk, pasta, pork, roots, vegetables, and legumes played smaller roles. Vegetable oil and sugar dominated the culinary ingredients group. Among processed foods, bread and cheese were the primary energy sources. Within the UPF group, margarine, cookies, snacks, breads, cold cuts, and sausages accounted for the greatest caloric contribution, while chocolates, ice cream, desserts, soft drinks, hot dogs, hamburgers, and sandwiches contributed less [7].

National adolescent dietary data reflect similar trends. A secondary analysis of the National School-Based Health Survey, including 159,245 adolescents, identified cookies, breads, and soft drinks as the most frequently consumed UPFs. Elevated intake was more prevalent among white female adolescents attending private schools and residing in non-capital cities [33].

These national patterns mirror broader global trends in which food environments shaped by physical, economic, political, and sociocultural dimensions exert a profound influence on food acquisition, preparation, and consumption [34]. Structural determinants, including race, income, educational attainment, and retail infrastructure such as store type, density, and geographic distribution, play a critical role in shaping dietary behaviors [35,36]. Limited access to outlets offering unprocessed or minimally processed foods has been consistently associated with a higher prevalence of obesity and metabolic disorders, particularly among socially and economically vulnerable populations [37,38,39].

Geographic areas with restricted access to affordable, nutrient-dense foods are known as “food deserts” [40]. Conversely, regions where food retail is dominated by UPF outlets are termed “food swamps” [41]. In the United States, food swamps show a stronger association with obesity than food deserts, particularly in communities with limited transportation options [41]. In Brazil, racial and socioeconomic inequalities exacerbate these conditions: Black individuals and individuals of mixed race report lower average incomes than white individuals, and healthier food outlets are more prevalent in affluent regions [34,42,43,44,45]. A longitudinal study of urban food environments from 2008 to 2020 revealed a reduction in food deserts alongside a rise in food swamps in a major Brazilian metropolis [46].

Together, these findings reinforce how structural inequities and profit-driven food environments converge to shape UPFs consumption patterns, amplifying metabolic risk and deepening public health disparities.

## 4. Obesity and Breast Cancer

Obesity can be defined using different criteria, including body mass index (BMI [kg/m^2^]) and patterns of fat distribution, particularly central versus peripheral adiposity. The latter is especially relevant, as it exerts a significant impact on metabolic health and hormonal signaling pathways [47]. More recently, an international commission convened experts to refine the definition and diagnostic criteria for obesity, characterizing it as a condition marked by excess adiposity, with or without abnormal distribution or dysfunction of adipose tissue, arising from multifactorial causes that remain incompletely understood [48].

Obesity is associated with an increased risk of developing several types of cancer, including endometrial, colorectal, and breast cancers [49]. In breast cancer specifically, the relationship between adiposity, cancer risk, and prognosis is complex and heterogeneous. Associations vary according to the timing of obesity onset, distinguishing premenopausal from postmenopausal obesity, as well as the menopausal status at cancer diagnosis. Furthermore, the impact of obesity on breast cancer risk differs according to tumor estrogen and progesterone receptor status and is influenced by the use of hormone replacement therapy in postmenopausal women [50].

In addition to its central role in metabolic disorders, excess adiposity has been causally linked to the development of at least thirteen malignancies, including postmenopausal breast cancer (BC) [51]. The association between obesity and BC, however, is complex and varies according to menopausal status and tumor subtype. Among premenopausal women, a higher body mass index (BMI) appears inversely associated with estrogen receptor-positive (ER+) tumors but positively associated with triple-negative disease. Conversely, in postmenopausal women, obesity markedly increases the risk of ER+ BC—particularly among those not receiving hormone replacement therapy—while exerting minimal influence on ER-negative tumors. Despite these subtype-specific differences in incidence, obesity consistently correlates with higher BC-specific and overall mortality across menopausal groups [52].

Quantitatively, in postmenopausal women, each 5-unit increase in BMI above 25 kg/m^2^ is associated with an approximate 10% elevation in BC risk [51]. The relationship is most pronounced for ER+ tumors [53]. Moreover, obesity has been associated with unfavorable clinicopathological features at diagnosis, including larger tumor size, lymph node involvement, and reduced survival. Collectively, these data suggest that obesity not only contributes to BC development but may also promote more aggressive tumor behavior and poorer clinical outcomes [52].

Mechanistically, chronic positive energy balance drives adipose tissue expansion, accompanied by hyperglycemia and compensatory hyperinsulinemia [54]. As adipocyte mass increases, adequate vascularization becomes necessary to sustain oxygen and nutrient delivery. This process depends on angiogenic signaling, particularly via vascular endothelial growth factor (VEGF) [21]. However, adipocytes possess limited lipid storage capacity. Progressive hypertrophy leads to local hypoxia, stabilizing hypoxia-inducible factor 1 alpha (HIF-1α), which in turn enhances VEGF expression, promotes extracellular matrix remodeling and fibrosis, and initiates inflammatory signaling within adipose depots [21,55,56,57]. Acting as an oxygen-sensitive transcription factor, HIF-1α upregulates leptin and VEGF while suppressing adiponectin expression, thereby contributing to a metabolically and hormonally dysregulated environment.

The inflammatory remodeling of obese adipose tissue is further amplified by adipocyte dysfunction and cell death. Leptin secretion increases and promotes recruitment of immune cells, leading to the formation of crown-like structures surrounding necrotic adipocytes [58]. Adipose tissue macrophages accumulate and undergo polarization toward a proinflammatory M1 phenotype. Free fatty acids released from dying adipocytes activate toll-like receptor 4 signaling pathways in both macrophages and adipocytes, triggering nuclear factor kappa B (NF-κB) activation and enhanced production of proinflammatory mediators, including tumor necrosis factor alpha, interleukin 6, interleukin 8, CCL2, CCL5, and VEGF [59]. These cytokines stimulate further lipolysis and aromatase expression, reinforcing NF-κB signaling and increasing local estrogen biosynthesis. Chemokines such as CCL2 sustain recruitment of additional monocytes, establishing self-perpetuating inflammatory circuits within obese adipose tissue [60].

Adaptive immune responses are also altered in obesity. Increased infiltration of CD8+ T cells and proinflammatory CD4+ Th1 and Th17 subsets—characterized by secretion of interferon gamma and interleukin 17A—occurs alongside reductions in Th2 and regulatory T-cell populations [52]. Enhanced presence of neutrophils, mast cells, B lymphocytes, and dendritic cells contributes to elevated production of inflammatory cytokines, whereas eosinophils, a source of anti-inflammatory IL-4 and IL-13, are diminished [59,60,61]. This collective shift favors a persistent proinflammatory immune landscape.

Although circulating estrogen levels decline sharply following menopause due to cessation of ovarian function [62], most BC cases are ER-positive and occur during the postmenopausal period [63]. This apparent paradox is partly explained by peripheral estrogen production within adipose tissue. Sensitive analytical techniques, including radioimmunoassays and liquid chromatography–tandem mass spectrometry, have demonstrated higher circulating estrogen concentrations in postmenopausal women with obesity compared to lean counterparts [54]. After menopause, adipose tissue becomes the primary site of estrogen synthesis through aromatase-mediated conversion of androgens. Aromatase expression increases in proportion to BMI and is particularly elevated in mammary adipose tissue [62,64,65]. These changes may drive estrogen-dependent tumor growth in susceptible tissues.

Finally, obesity-related tumor promotion reflects not only increased exposure to pro-tumorigenic mediators but also diminished protective signals. Levels of adiponectin—an adipokine with anti-inflammatory and insulin-sensitizing properties—are reduced in obesity, as are the potentially protective gut-derived hormones ghrelin and unacylated ghrelin [66,67]. The combined dysregulation of adipokines, sex steroids, metabolic factors, and inflammatory pathways establishes a biological environment highly permissive to breast cancer initiation and progression.

## 5. Obesity, Breast Cancer and Ultra-Processed Foods: Is There a Relationship?

The relationship between diet and cancer is considerably less linear than that observed between ultraviolet radiation and melanoma or between tobacco use and lung cancer [68]. The biological mechanisms through which ultra-processed foods may promote weight gain and obesity include excessive energy intake driven by heightened food reward arising from hyperpalatable nutrient combinations, as well as increased total energy intake and eating rate attributable to high energy density and specific orosensory properties such as soft texture and low fiber content [23].

Multiple biological mechanisms have been proposed to explain the metabolic consequences associated with ultra-processed food consumption. One pathway involves heightened activation of neural reward circuits, accompanied by disturbances in gut–brain communication that influence food valuation and decision-making. These effects may be driven by the high energy density of ultra-processed products and by the widespread use of artificial sweeteners, flavor enhancers, and color additives, which can modify sensory perception and post-ingestive signaling. In parallel, substantial alterations in gut microbiota composition and host–microbe interactions have been described. Such changes are thought to result from the structural degradation of whole-food matrices, the predominance of acellular nutrients, low dietary fiber content, and high loads of refined fats and sugars. The presence of food additives—including emulsifiers, non-nutritive sweeteners, and endocrine-disrupting chemicals—may further impair intestinal barrier integrity and metabolic regulation. Additionally, diets rich in ultra-processed foods are often relatively low in protein and fiber while containing large amounts of rapidly absorbable carbohydrates. This macronutrient profile may disrupt appetite regulation and satiety signaling, promoting excess energy intake. Together, these interconnected mechanisms contribute to impairments in glucose and lipid homeostasis that are increasingly linked to ultra-processed dietary patterns [23].

This obesogenic potential has been demonstrated experimentally. Under controlled conditions, individuals consuming UPFs exhibited significantly faster eating rates, measured both in kilocalories per minute and grams per minute, compared with those consuming minimally processed or fresh foods [69]. In solid form, ultra-processed foods tend to have high energy density due to their reduced fiber and water content, whereas in liquid form they commonly contain high levels of added sugars. When combined with accelerated eating speed and impaired satiety signaling, these characteristics substantially amplify total energy intake [70].

A recent study based on the UK Biobank demonstrated that high consumption of UPFs is associated not only with adverse metabolic profiles and increased adiposity, but also with structural alterations in subcortical brain regions involved in food intake regulation, including the nucleus accumbens, hypothalamus, putamen, pallidum, and amygdala. These microstructural brain changes were partially mediated by systemic inflammation, dyslipidemia, and body mass index, indicating that UPFs exert direct effects on the brain that extend beyond obesity mediated systemic pathways [71]. Collectively, this evidence indicates that UPFs influence neural circuits regulating eating behavior through mechanisms that are not fully explained by obesity alone.

These products elicit rapid glycemic responses while providing limited satiety. Specific industrial processes, including food matrix disruption, incorporation of additives, and texture softening, reduce mastication effort and enhance sensory appeal, thereby attenuating satiety signaling and promoting overconsumption. The high sugar, fat, and salt content of ultra-processed foods confers marked hyperpalatability, frequently displacing nutrient-dense foods and contributing to lower overall diet quality associated with weight gain [72].

Food processing also alters texture in ways that minimize chewing effort [73]. Reduced mastication has been associated with impaired satiety through neurohormonal mechanisms, including diminished activation of hypothalamic histaminergic pathways that are critical for appetite regulation [74]. Together, these mechanisms underscore that the impact of UPFs is not limited to their nutritional composition but also involves sensory and behavioral modifications that favor excessive energy intake.

Experimental evidence supports a role for mastication in metabolic regulation. In a controlled study, chewing food 40 times before swallowing, compared with 15 times, resulted in greater postprandial release of cholecystokinin and suppression of ghrelin levels [75]. Increased chewing reduces bolus particle size, enhances nutrient bioaccessibility, and amplifies gastrointestinal signaling pathways that promote satiety.

These mechanistic insights are supported by evidence from controlled clinical trials. In a crossover study involving 20 adults, participants consumed either an ultra-processed or an unprocessed diet ad libitum, both matched for total energy and macronutrient composition. The ultra-processed diet led to significantly higher energy intake and an average weight gain of 0.9 kg, whereas the unprocessed diet resulted in weight loss of similar magnitude. These differences have been partly attributed to factors such as faster eating rates and reduced chewing requirements associated with ultra-processed foods [76].

More recently, Dicken et al. [77] examined the impact of UPFs consumption within the context of national dietary guidelines by conducting a randomized crossover trial aligned with the United Kingdom Eatwell Guide. In this study, 55 adults received two ad libitum diets in random order: one based on minimally processed foods and the other composed predominantly of ultra-processed foods. The primary outcome was the within-participant difference in percentage weight change between diets. Both dietary patterns resulted in weight loss, with percentage weight change of −2.06% (95% confidence interval, −2.99 to −1.13) on the minimally processed diet and −1.05% (95% confidence interval, −1.98 to −0.13) on the ultra-processed diet. However, weight loss was significantly greater during the minimally processed diet, with a between-diet difference in percentage weight change of −1.01% (95% confidence interval, −1.87 to −0.14; *p* = 0.024). These findings indicate superior weight loss with minimally processed diets compared with ultra-processed diets and underscore the need to incorporate guidance on food processing into dietary recommendations, beyond existing nutrient-based guidelines [77].

These experimental findings are mirrored in population-based studies. In Brazil, a cross-sectional study of 218 women with breast cancer found that processed and ultra-processed foods accounted for nearly one-third of total caloric intake (28.4%) [78]. Individuals with abdominal obesity, as defined by waist circumference, reported higher consumption of more processed foods. Socioeconomic disparities were evident: women with incomplete primary education had significantly higher intake of these products than those with higher education levels. Importantly, regression models showed that a 5% increase in the caloric share of minimally processed foods was associated with a 3% reduction in the prevalence of overweight and abdominal obesity. In contrast, a 5% increase in the contribution of more processed foods was associated with 4% and 3% increases in the prevalence of overweight and abdominal obesity, respectively [78].

Ultra-processed foods (UPFs) contribute to cancer risk not only by promoting excess adiposity, a well-established risk factor for several malignancies, but also through direct exposure to dietary additives and processing derived contaminants, such as industrial trans fatty acids and acrylamide [79]. Evidence from the EPIC cohort, encompassing women from nine European countries, indicates that higher intake of industrial trans fatty acids, particularly elaidic acid, is associated with an increased risk of breast cancer. These findings suggest that, beyond their indirect effects mediated by adiposity, specific dietary components characteristic of ultra-processed foods may exert direct oncogenic effects [80].

Acrylamide was classified as a probable human carcinogen (Group 2A) by the International Agency for Research on Cancer in 1994. However, major concern emerged in 2002 following the identification of acrylamide formation in foods prepared at high temperatures above 120 °C under low-moisture conditions, particularly carbohydrate-rich foods subjected to baking or frying, such as French fries, bread, biscuits, and coffee [79]. To investigate the association between dietary acrylamide exposure and breast cancer risk, a prospective cohort study including 80,597 French women, with a mean follow-up of 8.8 years and stratification by menopausal and hormone receptor status, reported a positive association between dietary acrylamide intake and breast cancer risk, specifically among premenopausal women [81]. Similarly, a study conducted in the United Kingdom observed an increased breast cancer risk associated with higher acrylamide intake, with risk rising per 10 µg/day increment. In contrast, five prospective studies examining dietary acrylamide exposure and breast cancer risk reported null associations [82,83,84,85,86].

Taken together, these findings reinforce that dietary patterns characterized by high consumption of UPFs, particularly when combined with sedentary behavior, substantially contribute to excess adiposity, a well-established risk factor for breast cancer. The mechanisms underlying this association include unfavorable energy and nutrient profiles, disruption of food matrix integrity, impaired satiety signaling, excessive exposure to food additives and processing-derived contaminants, as well as behavioral and environmental drivers such as hyperpalatability, aggressive marketing, low cost, large portion sizes, and widespread product availability [11].

## 6. Ultra-Processed Foods, Gut Dysbiosis, and Breast Cancer

The gut microbiota orchestrates essential biological processes that maintain health and modulate disease susceptibility [87]. Comprising approximately 500 to 1000 bacterial species, the human gut is dominated by the Firmicutes, Bacteroidetes, Actinobacteria, and Proteobacteria phyla [88]. Within this complex ecosystem, genera such as Lactobacillus and Bifidobacterium produce metabolites like lactic acid, bacteriocins, and short-chain fatty acids (SCFAs), which enhance intestinal barrier integrity, increase secretory IgA, and regulate immune tolerance [89,90].

Mounting evidence suggests that the gut microbiota influences cancer susceptibility [91]. Diet plays a critical role in shaping the microbiome toward either tumor-suppressive or oncogenic profiles, depending on nutrient composition [92]. Commensal and symbiotic bacteria metabolize dietary components into bioactive molecules such as butyrate, a SCFA known to inhibit histone deacetylases and suppress tumorigenesis [93].

In women with breast cancer, significant gut dysbiosis has been documented, characterized by reduced abundance of Lactobacillus and Bifidobacterium species [87]. These alterations are associated with dysregulation of bacterial enzymes involved in estrogen metabolism, particularly β-glucuronidase, which mediates the deconjugation of estrogens in the gut. Altered β-glucuronidase activity can increase enterohepatic recycling of estrogens, thereby elevating systemic estrogen exposure and potentially contributing to increased breast cancer risk [94].

Diets rich in UPFs disrupt the gut microbial ecosystem, promoting dysbiosis marked by a decline in SCFA-producing taxa, increased intestinal inflammation, and impaired epithelial integrity [95]. An inflammatory milieu driven by microbial dysbiosis may impair antitumor immune surveillance while simultaneously facilitating angiogenesis, thereby contributing to tumor initiation and progression [96].

Beyond its local intestinal effects, the gut microbiota also influences systemic hormonal homeostasis, particularly through modulation of estrogen metabolism. This regulatory capacity is largely attributed to the “estrobolome,” a term describing the aggregate of microbial genes encoding enzymes involved in estrogen biotransformation [97]. Cancer development in this context reflects complex and dynamic crosstalk among the intestinal microbiota, the immune system, and tumor cells. Following hepatic conjugation, estrogens are excreted via bile into the gastrointestinal tract. Within the intestine, bacterial enzymes—including β-glucuronidases and β-glucosidases—can deconjugate these metabolites, restoring free estrogens that are subsequently reabsorbed into the circulation. This enterohepatic recirculation increases the pool of biologically active estrogens capable of binding to estrogen receptors, promoting cell cycle progression and stimulating tumor growth. In breast cancer, greater abundance of bacterial taxa characterized by elevated β-glucuronidase activity, such as *Clostridium coccoides*, *Clostridium leptum*, and *Blautia* species, has been associated with more advanced disease stages [98].

Nevertheless, findings from studies evaluating gut microbiota profiles in breast cancer remain inconsistent. While some investigations in postmenopausal women have reported increased alpha and beta diversity compared with healthy controls [99], others have observed reduced microbial diversity in affected individuals [100,101], and at least one study detected no significant differences in richness or overall community composition between groups [102]. These divergent results likely stem from variability in study design, sequencing platforms, analytical approaches, participant characteristics, and environmental or dietary exposures that are difficult to fully control.

## 7. Ultra-Processed Foods, Epigenetic Events, and Breast Cancer

Epigenetic marks do not alter the underlying DNA sequence. Instead, they modify the local chromatin environment, thereby influencing DNA accessibility and regulating a wide range of DNA-dependent processes, including gene transcription. Nutrients, toxins, pollutants, pesticides, and other environmental exposures can directly or indirectly affect the establishment, maintenance, and turnover of epigenetic marks. Obesity is, in part, epigenetically regulated, and obesity-associated epigenetic alterations have been linked to both excess adiposity and its downstream sequelae, contributing to the pathogenesis of multiple diseases, including breast cancer [49].

Epigenetic mechanisms represent an additional interface connecting obesity to breast cancer development, particularly through alterations in DNA methylation patterns and histone post-translational modifications. DNA methylation predominantly occurs at cytosine–phosphate–guanine (CpG) dinucleotides and regulates gene transcription without modifying the nucleotide sequence itself. In breast cancer tissues, aberrant methylation profiles have been consistently observed, characterized by promoter hypermethylation of tumor suppressor genes alongside hypomethylation of genes involved in oncogenic pathways, collectively facilitating malignant transformation [70]. Increased activity of DNA methyltransferases (DNMTs) plays a central role in this process, promoting transcriptional silencing of tumor suppressor loci [103,104]. Several critical genes—including *HOXA5*, *TMS1*, *p16*, *RASSF1A*, and *BRCA1*—as well as genes governing cell-cycle control, apoptosis, and estrogen signaling—exhibit abnormal promoter hypermethylation and reduced expression in breast cancer, reflecting dysregulated DNMT-mediated epigenetic control [103].

In addition to DNA methylation, histone modifications contribute substantially to epigenetic remodeling in breast carcinogenesis. Acetylation and methylation of specific amino acid residues within histone tails influence chromatin architecture and transcriptional accessibility. In breast cancer, shifts in these reversible modifications are associated with enhanced expression of oncogenes and repression of tumor suppressor genes, thereby supporting tumor growth, invasion, and metastatic potential [49]. Histone acetyltransferases catalyze the addition of acetyl groups to lysine residues on histones H3 and H4, promoting an open chromatin configuration and active transcription. Conversely, histone deacetylases remove these groups, favoring chromatin condensation and transcriptional suppression.

Histone methylation can occur in mono-, di-, or tri-methylated states, with functional consequences that depend on the specific residue modified, leading to either transcriptional activation or repression. Histone lysine methyltransferases are responsible for the addition of methyl groups, whereas histone demethylases remove these epigenetic marks [105]. Emerging evidence indicates that the biomechanical properties of the tumor microenvironment can influence histone acetylation patterns, thereby favoring tumor growth [106]. Consistently, in three-dimensional breast cancer models, histone deacetylase activity has been linked to increased tissue stiffness and enhanced tumorigenic potential [107].

Cancer arises not only from genetic mutations but also from epigenetic reprogramming that disrupts normal gene regulation. Environmental carcinogens, including those derived from dietary sources, can induce aberrations in cell cycle control by accumulating mutations, altering chromatin structure, and silencing tumor suppressor genes such as TP53 [105]. Disruption of histone modifications further contributes to carcinogenesis by promoting genomic instability, impaired DNA repair, and loss of checkpoint control [108].

Epigenetic alterations associated with UPFs consumption, particularly changes in DNA methylation, have emerged as important mediators of chronic disease risk [109]. In a study by Llauradó-Pont et al. [110], higher intake of ultra-processed foods was associated with differential methylation at seven CpG sites, including hypermethylation of cg14665028 located within the promoter region of the NHEJ1 gene. This epigenetic modification has been linked to increased DNA damage in mice fed a high-fat diet, supporting the existence of a diet–epigenome–cancer axis [110,111].

In this sense, we hypothesized that chronic consumption of UPFs may promote a pro-tumorigenic epigenetic state, characterized by hypermethylation of tumor suppressor genes and chromatin dysregulation, thereby contributing to the initiation and progression of breast cancer.

## 8. Ultra-Processed Foods, Inflammation and Breast Cancer

In the epigenetics section that the discussion focuses on the most commonly investigated epigenetic mechanisms reported in the literature, particularly DNA methylation and histone modifications.

A growing body of evidence links ultra-processed food (UPF) consumption to obesity and its downstream metabolic consequences, including persistent low-grade inflammation and impaired immune regulation. The progressive global adoption of Western-style dietary patterns—characterized by a high proportion of industrially formulated products enriched with additives and emulsifiers—has intensified exposure to these components. Such substances can alter gut microbial ecology and functionality, compromise intestinal barrier integrity, and induce immune-related epigenetic modifications. These disturbances may facilitate metabolic endotoxemia and sustain chronic systemic inflammation [112].

Dietary contributions to inflammation are not restricted to microbiota-dependent mechanisms. Advanced glycation end products (AGEs) and advanced lipoxidation end products (ALEs), generated during industrial processing or high-temperature, low-moisture cooking methods, are absorbed through the diet and have been associated with heightened appetite, excessive caloric intake, and subsequent weight gain. Their accumulation is also linked to oxidative stress and inflammatory activation [113]. Furthermore, foods with elevated glycemic load—such as refined grains and isolated sugars commonly present in UPFs—promote rapid fluctuations in blood glucose and insulin levels, enhancing oxidative stress and triggering transcription of proinflammatory genes and signaling pathways [114].

Several additional nutritional factors may further promote inflammation and potentially contribute to the development of low-grade systemic inflammation. These include deficiencies in key micronutrients, such as zinc and magnesium, which may arise from diets high in UPFs that are typically poor in vitamins and minerals, as well as suboptimal omega-3 fatty acid status, which impairs the resolution phase of inflammation [115,116,117].

This persistent inflammatory state promotes malignant transformation and tumor initiation in surrounding tissues, primarily through sustained release of growth factors and reactive oxygen and nitrogen species. These mediators induce DNA damage in proliferating epithelial cells and contribute to the establishment of irreversible genomic instability. Beyond tumor initiation, inflammation also plays a critical role in tumor promotion, malignant progression, and metastatic dissemination. These processes are driven by a complex network of cytokines, chemokines, prostaglandins, free radicals, growth factors, and proteolytic enzymes produced by a heterogeneous cellular compartment within the tumor microenvironment, including macrophages, neutrophils, lymphocytes, dendritic cells, natural killer cells, fibroblasts, adipocytes, and endothelial cells [118].

Mounting evidence indicates that UPFs may act as upstream modulators of inflammatory pathways implicated in carcinogenesis. The dietary inflammatory index has proven useful in elucidating diet–inflammation–cancer relationships, with higher index scores consistently associated with increased cancer risk [119,120]. Notably, nutrients such as palmitic acid, which is abundant in many UPFs, disrupt endoplasmic reticulum and mitochondrial function, thereby promoting cellular stress responses and inflammation [121]. In addition, excessive caloric intake derived from UPFs supports adipose tissue expansion, perpetuating a state of low-grade systemic inflammation. Saturated fatty acids further activate toll-like receptors, particularly TLR2 and TLR4, triggering canonical inflammatory signaling cascades, including the NF-κB pathway [122].

Diet-induced dysbiosis further amplifies this proinflammatory state. Although interindividual variability remains a challenge, converging mechanistic and epidemiological evidence increasingly supports a strong association between UPFs consumption, systemic inflammation, and breast cancer pathogenesis [123].

## 9. Ultra-Processed Foods, Metabolic Disorders, and Breast Cancer

The marked worldwide increase in ultra-processed food (UPF) consumption has coincided with—and may have contributed to—the growing burden of noncommunicable chronic diseases, particularly obesity [124]. Sustained positive energy balance promotes adipose tissue expansion, hyperglycemia, and compensatory hyperinsulinemia. These metabolic disturbances are accompanied by systemic and local alterations that collectively establish a tumor-permissive microenvironment [54].

Breast cancer progression is influenced by the disruption of interconnected metabolic signaling networks. Obesity-associated mediators—including insulin, leptin, hypoxic signaling, and inflammatory factors such as prostaglandin E2 (PGE2), tumor necrosis factor (TNF), and interleukin 6 (IL-6)—modulate pathways operating within both malignant cells and surrounding stromal compartments [54]. In tumor cells, insulin, leptin, and estradiol converge to activate the phosphoinositide 3-kinase (PI3K)–AKT axis, while leptin and estradiol attenuate AMP-activated protein kinase (AMPK) activity. Concurrently, hypoxic conditions stabilize hypoxia-inducible factor 1 alpha (HIF-1α), reinforcing a metabolic shift toward aerobic glycolysis. This reprogramming enhances glucose uptake, nucleotide and protein biosynthesis, and ultimately supports rapid cellular proliferation [125].

Within adipose stromal cells, similar mediators exert complementary effects. Leptin, PGE2, TNFα, and IL-6 increase glucose transporter expression and facilitate glucose influx. In parallel, leptin and PGE2 promote HIF-1α signaling, suppress the LKB1–AMPK pathway, inhibit p53 function, and upregulate aromatase—the rate-limiting enzyme responsible for estrogen synthesis. The cumulative outcome is expansion of stromal-derived cellular populations capable of producing lactate and estrogens, metabolites that further fuel tumor growth and reinforce metabolic reprogramming in adjacent cancer cells [126].

Adipose tissue functions as a metabolically active endocrine organ composed of adipocytes and diverse stromal cell populations, including endothelial cells, pericytes, macrophages, and adipocyte progenitors. In breast cancer, adipocyte-rich microenvironments play a pivotal role in tumor progression by supplying metabolic substrates and secreting signaling molecules that support tumor growth and survival [127].

Collectively, these findings elucidate the metabolic mechanisms linking obesity and UPFs consumption to breast cancer development and progression, while also identifying potential therapeutic entry points. These range from dietary and lifestyle interventions to strategies targeting metabolic reprogramming and inflammatory signaling pathways within the tumor microenvironment.

## 10. Ultra-Processed Foods, Oxidative Stress and Breast Cancer

Oxidative stress arises from an imbalance between the generation of reactive oxygen species and the capacity of endogenous antioxidant defense systems. This pro-oxidative state promotes molecular damage to DNA, proteins, and lipids, and when such damage is not adequately repaired, it can lead to mutations that initiate and promote carcinogenesis [128]. Owing to their high reactivity, reactive oxygen species can modify biomolecular structures, alter cellular localization, disrupt intermolecular interactions, and impair normal biological functions.

Persistent positive energy balance generates a metabolic surplus that drives activation of lipogenic programs and progressive expansion of adipose tissue. Diets dominated by ultra-processed foods frequently contain elevated levels of saturated fatty acids, which further potentiate lipid accumulation [129]. Saturated fatty acids activate peroxisome proliferator–activated receptor gamma (PPARγ), a transcription factor that upregulates genes encoding lipogenic enzymes and promotes adipocyte lipid storage [130]. Enhanced lipogenesis increases triacylglycerol synthesis within the endoplasmic reticulum, predisposing adipocytes to endoplasmic reticulum stress and activation of the unfolded protein response. This adaptive pathway, when chronically engaged, stimulates transcriptional networks linked to oxidative stress and inflammation, including nuclear factor kappa B (NF-κB), NADPH oxidase, and inducible nitric oxide synthase signaling cascades [131]. The resulting elevation in intracellular reactive oxygen species amplifies the secretion of proinflammatory cytokines by adipocytes, reinforcing a pro-oxidative and inflammatory tissue environment [132].

Progressive triacylglycerol accumulation also leads to adipocyte hypertrophy, which—together with oxidative stress—facilitates recruitment and polarization of proinflammatory M1 macrophages. In obesity, macrophages may account for up to 40% of total adipose tissue cellularity. These adipose tissue macrophages contribute to extracellular matrix remodeling and sustained production of inflammatory mediators, displaying functional heterogeneity and substantial metabolic reprogramming that perpetuate local and systemic inflammation [133].

Notably, individuals with higher consumption of UPFs exhibit reduced activity of key antioxidant enzymes, such as catalase (CAT) and superoxide dismutase (SOD), together with elevated levels of xanthine oxidase [134,135]. This enzymatic profile reflects an impaired antioxidant defense and an increased capacity for reactive oxygen species generation, thereby reinforcing the oxidative burden associated with diets rich in UPFs.

Oxidative stress plays a fundamental role throughout the multistep process of carcinogenesis. In early tumor initiation, excessive generation of reactive oxygen species (ROS) induces DNA damage, leading to mutational events affecting both oncogenes and tumor suppressor genes. Beyond direct genomic injury, ROS also modify redox-sensitive amino acid residues—particularly cysteine and methionine—within regulatory proteins. These oxidative alterations can disrupt protein conformation and enzymatic function, thereby perturbing critical signaling cascades such as RAS–MEK–ERK1/2, PI3K–AKT, Keap1–ARE, NF-κB, and JAK–STAT. As tumor development advances, persistent oxidative imbalance sustains genomic instability and reinforces aberrant proliferative signaling. This cumulative redox-driven damage enhances cellular plasticity, promoting invasive behavior and metastatic dissemination during later stages of cancer progression [128].

**Figure 2:**
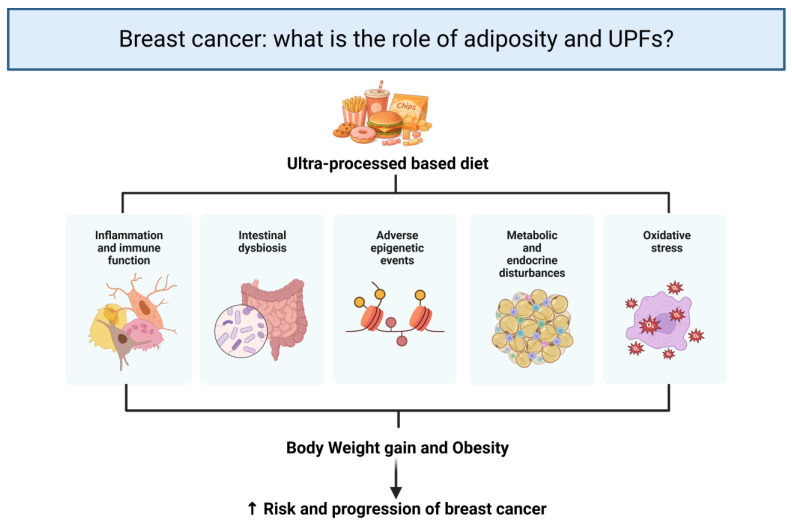
Proposed mechanisms linking ultra-processed food (UPFs) consumption to breast cancer risk and progression. An ultra-processed food–based diet may promote systemic inflammation and immune dysfunction, intestinal dysbiosis, adverse epigenetic modifications, metabolic and endocrine disturbances, and increased oxidative stress. These interconnected pathways contribute to body weight gain and obesity, which in turn are associated with an increased risk and progression of breast cancer. Created in BioRender. Torres-Leal, L. (2026) https://biorender.com/1w8t6ld.

Figure 2 summarizes the complex mechanisms through which UPFs consumption may influence breast cancer development. These mechanisms include alterations in the gut microbiota and its metabolites, some of which may exert oncogenic or tumor-suppressive effects, DNA damage and accumulation of somatic mutations, chronic inflammation, immune dysregulation, metabolic and hormonal disturbances such as insulin resistance, and oxidative stress. Collectively, these interconnected processes contribute to the initiation and progression of breast cancer [136,137].

## 11. Limitations of Review

This review has several limitations that should be acknowledged. First, as a narrative review, the present study does not follow the structured methodological framework of a systematic review, which may introduce selection bias and limit reproducibility. In addition, no formal risk-of-bias assessment was performed, which restricts the ability to compare the methodological quality of the included studies. Another important limitation relates to the heterogeneity of the evidence base, which includes preclinical experiments, observational human studies, and review articles. Furthermore, much of the epidemiological evidence relies on observational designs, which are inherently susceptible to residual confounding and measurement errors in dietary assessment. Finally, several mechanistic pathways discussed are primarily supported by experimental models, and further clinical and translational research is needed to confirm their relevance in human breast cancer.

## 12. Conclusions

The relationship between UPFs consumption, obesity, and breast cancer is increasingly supported by robust scientific evidence. Excessive intake of UPFs substantially contributes to weight gain and the development of obesity, a well-established risk factor for breast cancer, particularly among postmenopausal women. Beyond their obesogenic effects, specific components of UPFs, including additives and low-quality nutrient profiles, may directly modulate key biological mechanisms implicated in breast carcinogenesis, such as chronic inflammation, oxidative stress, hormonal dysregulation, and epigenetic alterations.

Taken together, these findings position UPFs consumption as a critical and modifiable upstream determinant of breast cancer risk, with clear implications for public health strategies. Addressing UPFs exposure extends beyond individual dietary choices and requires population-level interventions that reshape food environments, improve access to fresh and minimally processed foods, and reduce structural drivers of unhealthy dietary patterns.

Integrating dietary guidance that prioritizes natural and minimally processed foods into cancer prevention frameworks represents a feasible and evidence-informed approach to mitigating obesity-related cancer risk. Such guidance should be incorporated into national dietary guidelines, cancer prevention programs, and clinical counseling, while being reinforced by regulatory measures, including front-of-package labeling, restrictions on marketing of UPFs, fiscal policies, and food system reforms. These strategies have the potential to reduce chronic inflammation, metabolic dysfunction, and hormonal dysregulation at the population level, thereby contributing to meaningful reductions in breast cancer incidence and the broader burden of noncommunicable diseases.

## Data Availability

No new data were created or analyzed in this study. Data sharing is not applicable.
